# Succinic acid production by wine yeasts and the influence of GABA and glutamic acid

**DOI:** 10.1007/s10123-023-00410-9

**Published:** 2023-07-27

**Authors:** Rafael Torres-Guardado, Nicolás Rozès, Braulio Esteve-Zarzoso, Cristina Reguant, Albert Bordons

**Affiliations:** 1 Grup de Biotecnologia Enològica, Departament de Bioquímica i Biotecnologia, Universitat Rovira i Virgili, Facultat d’Enologia, C/ Marcel·lí Domingo 1, 43007 Tarragona, Catalonia Spain; 2 Grup de Biotecnologia Microbiana dels Aliments, Departament de Bioquímica i Biotecnologia, Universitat Rovira i Virgili, Facultat d’Enologia, C/ Marcel·lí Domingo 1, 43007 Tarragona, Catalonia Spain

**Keywords:** GABA, Glutamic acid, Non*-Saccharomyces*, Succinic acid, Yeast, Wine

## Abstract

As a consequence of alcoholic fermentation (AF) in wine, several compounds are released by yeasts, and some of them are linked to the general quality and mouthfeel perceptions in wine. However, others, such as succinic acid, act as inhibitors, mainly of malolactic fermentation. Succinic acid is produced by non-*Saccharomyces* and *Saccharomyces* yeasts during the initial stages of AF, and the presence of some amino acids such as γ-aminobutyric acid (GABA) and glutamic acid can increase the concentration of succinic acid. However, the influence of these amino acids on succinic acid production has been studied very little to date. In this work, we studied the production of succinic acid by different strains of non-*Saccharomyces* and *Saccharomyces* yeasts during AF in synthetic must, and the influence of the addition of GABA or glutamic acid or a combination of both. The results showed that succinic acid can be produced by non-*Saccharomyces* yeasts with values in the range of 0.2–0.4 g/L. Moreover, the addition of GABA or glutamic acid can increase the concentration of succinic acid produced by some strains to almost 100 mg/L more than the control, while other strains produce less. Consequently, higher succinic acid production by non-*Saccharomyces* yeast in coinoculated fermentations with *S. cerevisiae* strains could represent a risk of inhibiting *Oenococcus oeni* and therefore the MLF.

## Introduction

Winemaking is a complex process that usually involves two important microbiological steps: alcoholic fermentation (AF) carried out by yeasts, which produce ethanol and carbon dioxide by glucose breakdown (Fleet 1998; Ribéreau-Gayon et al. 2006), and malolactic fermentation (MLF) carried out by lactic acid bacteria (LAB), where L-malic acid is decarboxylated to produce L-lactic acid (Liu 2002; Bartowsky 2005). Besides ethanol and CO_2_, yeasts produce other compounds linked to quality and mouthfeel perception, such as polyols, esters and other alcohols (Dicks et al. 1995; Fleet 2003; Bartowsky and Pretorius 2009; Styger et al. 2011). Nonetheless, other compounds such as medium-chain fatty acids (MCFA) and organic acids are also produced (Guilloux-Benatier et al. 1998; Balmaseda et al. 2018).

**Fig. 1:**
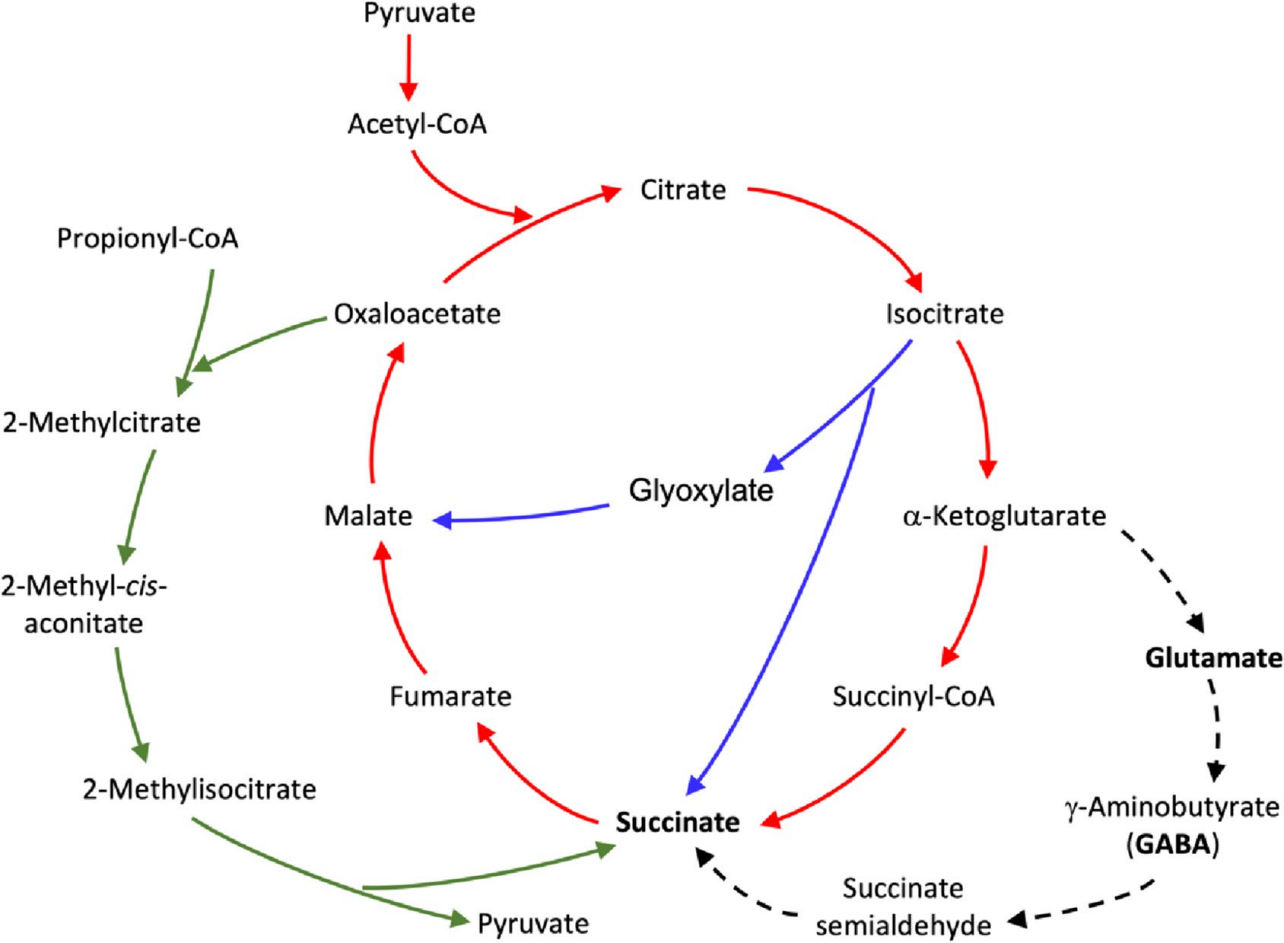
Metabolic pathways of yeasts and many other organisms where succinate acts as an intermediary: tricarboxylic acid cycle (TCA) (red lines), glyoxylate shunt (blue lines), methylcitric acid cycle (green lines) and GABA shunt (black dashed lines). Adapted from De Klerk (2010) and Freitas e Silva et al. (2020)

Succinic acid is an organic acid produced by yeasts during the early stages of alcoholic fermentation (De Klerk 2010) and acts as an intermediary in important metabolic pathways, such as the tricarboxylic acid cycle (TCA), glyoxylic acid shunt, methylcitric acid cycle and GABA shunt (Fig. 1). Moreover, this acid can improve the sensory wine properties due to the increase in fruity aromatic esters such as methyl-succinate, ethyl-succinate and diethyl succinate (Jordán et al. 2002).

*Saccharomyces cerevisiae* strains are known to produce succinic acid at levels of 200 mg/L to 2 g/L (Coulter and Pretorius 2007; De Klerk 2010; Zhu et al. 2020). Non-*Saccharomyces* yeasts can also produce succinic acid, usually in a range between 0.3 and 0.95 g/L (Ciani and Maccarelli 1997; Escribano et al. 2018; Zhu et al. 2020). Moreover, Contreras et al. (2014) found that some strains of *Metschnikowia pulcherrima*, *Schizosaccharomyces malidevorans* and *Candida stellata* produced 1 to 2 g/L of succinic acid*.*

In this sense, we must consider that there is an increasing oenological interest in these non-*Saccharomyces* yeasts (Padilla et al. 2016) due to the production of new aromas (Belda et al. 2017), and currently, an increasing number of cellars are using them, often inoculating with them prior to *S. cerevisiae*.

Succinic acid can have a positive impact on the general quality of wine, as mentioned above, due to ester formation. In contrast, this acid can have a negative impact on LAB development and consequently on MLF performance because it has been reported that it can act as an inhibitor of L-malic decarboxylation in LAB (Lonvaud-Funel and Strasser de Saad 1982; Son et al. 2009). Moreover, succinic acid can inhibit MLF performance and *Oenococcus oeni* strains (Caridi and Corte 1997; Torres-Guardado et al. 2022).

There are many factors that influence succinic acid production by yeast, such as temperature, assimilable nitrogen, oxygen and amino acids (De Klerk 2010). Indeed, succinic acid levels in red wines have been reported to be twice that of white wines (Coulter and Pretorius 2007). This difference could be explained by the increase in GABA in the grape must due to maceration of red grapes, which could favour succinic acid production by yeast (De Klerk 2010).

Therefore, due to the importance of succinic acid production by yeasts, the increasing use of non-*Saccharomyces* strains and the impact of succinic acid on MLF, the aim of this work was to evaluate the influence of GABA and glutamic acid supplementation on succinic acid production by non-*Saccharomyces* yeasts in synthetic must.

## Materials and Methods

### Yeast strains and culture conditions

Twelve non-*Saccharomyces* strains and two *S. cerevisiae* strains were used in this study (Table 1). Before the fermentation assays, 10^6^ cells/mL of each strain were precultured in 12 mL YPD broth (20 g/L of dextrose, 20 g/L of peptone and 10 g/L of yeast extract (Cultimed, Barcelona, Spain)). Yeasts were incubated at 28 °C for 72 h at least twice before experimental use.

**Table 1:** Yeast strains used in this study

Strain code	Species	Source	Strain full name
ScK1	*Saccharomyces cerevisiae*	Lallemand Inc	Lalvin ICV K1 Marquée™
ScQA23	*Saccharomyces cerevisiae*	Lallemand Inc	Lalvin QA23™
TdBio	*Torulaspora delbrueckii*	Lallemand Inc	Level 2 Biodiva™
TdNS	*Torulaspora delbrueckii*	Agrovin S.A	Viniferm NSTD
TdZym	*Torulaspora delbrueckii*	Laffort ^R^	Zymaflore^R^ Alpha
Td13135	*Torulaspora delbrueckii*	Padilla et al. 2017	CECT 13135
MpFla	*Metschnikowia pulcherrima*	Lallemand Inc	Flavia™
Mp13131	*Metschnikowia pulcherrima*	Padilla et al. 2017	CECT 13131
Hu10389	*Hanseniaspora uvarum*	CECT	CECT 10389
Hu13130	*Hanseniaspora uvarum*	Padilla et al. 2017	CECT 13130
HvT02	*Hanseniaspora vineae*	Medina et al. 2018	T02/05F
Hv1471	*Hanseniaspora vineae*	CECT	CECT 1471
Sb11109	*Starmerella bacillaris*	CECT	CECT 11109
Sb13129	*Starmerella bacillaris*	Padilla et al. 2017	CECT 13129

*CECT: Spanish Type Culture Collection

### Fermentations

Alcoholic fermentations were carried out in synthetic must recently developed by Ruiz-de-Villa et al. (2023) containing 110 g/L glucose, 110 g/L fructose, 5 g/L L-tartaric acid, 2 g/L L-malic acid, 0.5 g/L citric acid, 1.7 g/L yeast nitrogen base w/o amino acids, 50 mg/L NH_4_Cl and 1.505 g/L amino acid stock at pH 3.5. This amino acid stock contained, among others, 50 mg/L GABA and 210 mg/L glutamic acid. In addition to this control, three different treatments were performed, where the content of these two amino acids was increased: 100 mg/L GABA, 420 mg/L glutamic acid, and 100 mg/L GABA + 420 mg/L glutamic acid. All fermentations were performed in triplicate in 100 mL of synthetic must in 150 mL bottles at 22 °C. The different yeasts were inoculated at 1 × 10^6^ cells/mL. Samples were taken at 48 h to evaluate the succinic acid production and sugar consumption rate, following strategy of Martín-García et al. (2020).

### Chemical analyses

The succinic acid content in the final samples of the AF trials was determined by high-performance liquid chromatography (HPLC) following the method of Zhu et al. (2020). All samples were previously filtered by injecting them through 0.2 μm Captiva filters (Agilent Technologies, Santa Clara CA, USA). The chromatograms were analysed using Agilent ChemStation Plus software.

The sugar (glucose + fructose) consumption rate was calculated from the decrease in density (g/cm^3^ at 20 °C) from 0 to 48 h and converting it to a sugar concentration, following the recommended conversion of OIV (2022).

### Statistical analyses

The data obtained were subjected to analysis of variance (ANOVA)s using Fisher’s least significant difference (LSD) and Tukey’s tests. XLSTAT 2020.2.3 software (Addinsoft, Paris, France) was used and the confidence interval was 95%, obtaining significative results with a probability (p) value of ≤ 0.05.

## Results and Discussion

### Succinic acid production by wine yeasts

The production of succinic acid was evaluated in control treatments (the synthetic must contained 50 mg/L GABA and 210 mg/L glutamic acid) at 48 h to determine the total amount produced by every strain. Although the AF were not still finished at 48 h (data not shown), we chose this sampling time because it is known that most succinate is formed from pyruvate by yeasts in the first days of AF (Ribéreau-Gayon et al. 2006), and also because the usual cellar strategy when using non-*Saccharomyces* yeasts is inoculating them first, followed by inoculation with *S. cerevisiae* after 48 h (Martín-García et al. 2020).

**Fig. 2:**
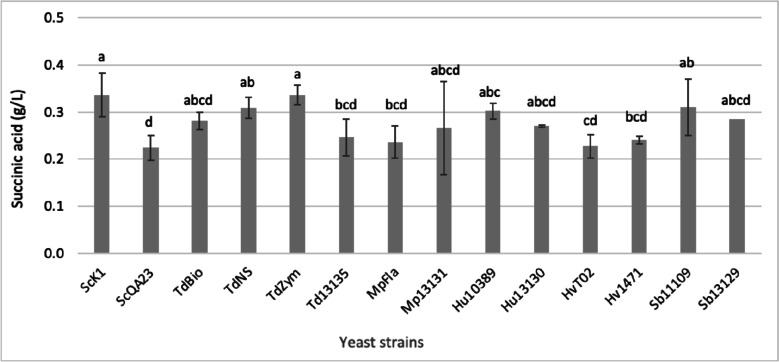
Succinic acid production by different yeast strains (see codes in Table 1) in synthetic must. Values shown are the mean and standard deviation (SD) of triplicates. Different subscript letters (^a−d^) indicate that yeast treatments are significantly different at *p* ≤ 0.05 according to Tukey’s post hoc comparison test

The results showed that the TdZym and ScK1 strains produced approximately 0.34 g/L (Fig. 2), the highest concentration of succinic acid compared with the other strains, in which the HvT02 and ScQA23 strains produced approximately 0.22 g/L. However, for the other strains used in this experiment, there were no significant differences in terms of succinic acid production, ranging between 0.23–0.30 g/L. Other studies reported succinic acid in synthetic must that ranged between 0.3–2.0 g/L (Ciani and Maccarelli 1997; Contreras et al. 2014).

As seen, in some cases there were significant differences in succinic acid production among strains of the same species (Fig. 2). This variability is probably related with the wide biodiversity found for other metabolic characteristics in wine yeasts, as has been mentioned by other authors (Romano et al 2008). Variability also depends on whether it is a commercial strain or a wild one. Obviously, this subject requires further investigation, looking for it in several strains of each species, taking in account their source and isolation place, and looking for possible intraspecific genetic differences. Considering the differences among strains, we also calculated the average values of this acid production for each species (Table 2) in order to see possible significant differences between species. As seen Table 2, two non-*Saccharomyces* species, *H. uvarum* and *S. bacillaris,* produced significantly more succinic acid than the others, with values higher than 0.3 g/L, while *T. delbrueckii* and *M. pulcherrima* produced succinic acid levels like those of *S. cerevisiae*. This is an interestingly positive result since *T. delbrueckii* and *M. pulcherrima* are currently the most used non-*Saccharomyces* species in winemaking (Balmaseda et al. 2021). Escribano et al. (2018) found similar values of succinic acid produced by *T. delbrueckii* in real wines but higher values for *M. pulcherrima* —around 0.62 g/L— and slightly higher values for *S. cerevisiae* —around 0.45 g/L.

**Table 2:** Succinic acid production by different yeast species in synthetic must. Values shown are the mean values for different strains that were combined for each species. ^a−c^ Values are significantly different at *p* ≤ 0.05 according to a Anova Fisher’s least significant difference test

Species	Succinic acid (g/L)
*Saccharomyces cerevisiae*	0.252 b
*Torulaspora delbrueckii*	0.267 b
*Metschnikowia pulcherrima*	0.269 b
*Hanseniaspora uvarum*	0.300 a
*Hanseniaspora vineae*	0.220 c
*Starmerella bacillaris*	0.315 a

Additionally, it must be considered that in some cases the AF period can be longer than 48 h, resulting in higher amounts of succinic acid produced by non-*Saccharomyces* yeasts. Moreover, the succinic acid levels produced before the sequential inoculation of *S. cerevisiae* could be increased through AF after its inoculation, since sometimes *S. cerevisiae* by itself can produce until 2 g/L of succinic acid (Caridi and Corte 1997; De Klerk 2010).

Consequently, succinic acid production by using non-*Saccharomyces* yeasts in combination with *S. cerevisiae* could represent a risk of stalling MLF because this acid can act as an inhibitor of *O. oeni*. In a previous work, we showed that this acid can exert an inhibitory effect on *O. oeni*, and consequently on MLF development, at concentrations higher than 1 g/L and this inhibition was higher at pH 3.5 than at pH 4.0. This negative effect was more evident when succinic acid was at a molar concentration higher than that of L-malic acid (Torres-Guardado et al. 2022).

### Effect of GABA and glutamic acid addition on sugar consumption by yeast strains

In addition to examining succinic acid production, we also evaluated the influence of GABA, glutamic acid, and a combination of the two, when added to the synthetic must, on the development of AF by the different yeast strains. For that purpose, we used the sugar consumption rate obtained during the first 48 h (Table 3). The addition of GABA and glutamic acid exerted slight inhibition on the sugar consumption rate in most strains. However, in some strains, such as TdNS, MpFla, Mp13131, HvT02 and Sb11109, the sugar consumption rate was not affected relative to the control. On the other hand, only two strains (ScK1 and Hv1471) presented increased sugar consumption rate upon amino acid addition to the medium, with a significant increase in the sugar consumption rate of almost 0.1 mg/mL/h (Table 3). Considering the species, we see an inhibition of sugar consumption in most *T. delbrueckii* and *H. uvarum* strains when GABA and/or glutamic acid were added, but there was no variation of sugar consumption in the *M. pulcherrima* strains.

**Table 3:** Sugar consumption rate by different yeast strains (see Table 1 for codes) in synthetic must with increased concentration of GABA, glutamic acid (Glut) or a combination of both. Values shown are the mean and standard deviation (SD) of triplicates

	Sugar consumption rate (mg/mL/h)			
Strain	Control *	100 mg/L GABA	420 mg/L Glut	100 mg/L GABA and 420 mg/L Glut
ScK1	0.771 ± 0.026 b	0.938 ± 0.022 a	0.938 ± 0.022 a	0.972 ± 0.022 a
ScQA23	0.656 ± 0.022 a	0.486 ± 0.018 b	0.542 ± 0.018 b	0.549 ± 0.018 b
TdBio	0.552 ± 0.022 a	0.340 ± 0.018 b	0.333 ± 0.018 b	0.354 ± 0.018 b
TdNS	0.448 ± 0.022 a	0.417 ± 0.018 a	0.410 ± 0.018 a	0.389 ± 0.018 a
TdZym	0.510 ± 0.013 a	0.424 ± 0.011 b	0.396 ± 0.011 b	0.403 ± 0.011 b
Td13135	0.385 ± 0.013 a	0.326 ± 0.011 b	0.319 ± 0.011 ab	0.333 ± 0.011 b
MpFla	0.260 ± 0.010 a	0.250 ± 0.008 a	0.257 ± 0.008 a	0.271 ± 0.008 a
Mp13131	0.354 ± 0.024 a	0.292 ± 0.020 a	0.278 ± 0.020 a	0.285 ± 0.020 a
Hu10389	0.344 ± 0.020 a	0.250 ± 0.017 b	0.229 ± 0.017 b	0.257 ± 0.017 b
Hu13130	0.344 ± 0.013 a	0.250 ± 0.011 b	0.222 ± 0.011 b	0.264 ± 0.011 b
HvT02	0.542 ± 0.032 a	0.549 ± 0.027 a	0.507 ± 0.027 a	0.535 ± 0.027 a
Hv1471	0.427 ± 0.011 b	0.514 ± 0.009 a	0.521 ± 0.009 a	0.535 ± 0.009 a
Sb11109	0.354 ± 0.017 a	0.368 ± 0.014 a	0.333 ± 0.014 a	0.340 ± 0.014 a
Sb13129	0.417 ± 0.011 a	0.361 ± 0.009 b	0.347 ± 0.009 b	0.361 ± 0.009 b

^a−b^Values for each strain in the same row followed by different letters are significantly different at *p* ≤ 0.05 according to a Tukey post-hoc test

*Control was synthetic must containing 50 mg/L GABA and 210 mg/L glutamic acid

Nitrogen assimilation in yeasts is controlled by nitrogen catabolite repression (NCR) and some species or strains prefer to assimilate N sources in different orders (Gobert et al. 2017). Therefore, we supposed that the decrease in the sugar consumption rate of some strains when concentrations of GABA and glutamic acid were increased, may be a consequence of regulation of nitrogen metabolism. This could have affected other major pathways, such as sugar and sulphur metabolism, which can led to the production of active intermediates, flavours and end-products (Hirst and Richter 2016). In contrast, it is remarkable that ScK1 strain showed a significant increase in the C-source consumption rate (Table 3), while other *non-Saccharomyces* yeasts were not affected by increased concentrations of GABA and glutamic acid. Therefore, there is a strain-dependent effect, since variability occur within yeast species, as indicated by Kemsawasd et al. (2015).

### Effect of GABA and glutamic acid on succinic acid production

The addition of GABA or glutamic acid or both can differentially affect succinic acid production in different yeast strains (Table 4). For example, in contrast with the control assay (synthetic must containing 50 mg/L GABA and 210 mg/L glutamic acid), in the experiments where glutamic acid was increased, TdBio, HvT02 and Hv1471 produced almost 50 mg/L more succinic acid. Moreover, this effect was also observed when GABA was added for the same strains HvT02 and Hv1471, which produced approximately 80 mg/L more succinic acid than the control. In contrast, TdBio, Hu10389, Hu13130 and Sb11109 showed a decrease of approximately 50–150 mg/L when both GABA and glutamic acid were added, and this effect was also observed for Hu13130 in the medium where only GABA was increased, which suggests that GABA is responsible for the decrease in succinic acid production.

**Table 4:** Succinic acid production by different yeast strains in synthetic must with increased concentration of GABA, glutamic acid (Glut) or a combination of both. Values shown are the mean and standard deviation (SD) of triplicates

	Succinic acid (g/L)			
Strain	Control *	100 mg/L GABA	420 mg/L Glut	100 mg/L GABA and 420 mg/L Glut
ScK1	0.338 ± 0.016 a	0.304 ± 0.013 a	0.301 ± 0.013 a	0.297 ± 0.013 a
ScQA23	0.227 ± 0.029 ab	0.155 ± 0.029 b	0.278 ± 0.029 a	0.192 ± 0.029 ab
TdBio	0.296 ± 0.014 b	0.207 ± 0.012 c	0.344 ± 0.012 a	0.208 ± 0.012 c
TdNS	0.299 ± 0.045 a	0.259 ± 0.037 a	0.327 ± 0.037 a	0.248 ± 0.037 a
TdZym	0.347 ± 0.034 ab	0.347 ± 0.028 ab	0.373 ± 0.028 a	0.253 ± 0.028 b
Td13135	0.235 ± 0.027 a	0.173 ± 0.022 a	0.183 ± 0.022 a	0.185 ± 0.022 a
MpFla	0.237 ± 0.017 a	0.266 ± 0.014 a	0.277 ± 0.014 a	0.258 ± 0.014 a
Mp13131	0.258 ± 0.014 b	0.293 ± 0.011 ab	0.281 ± 0.011 ab	0.313 ± 0.011 a
Hu10389	0.335 ± 0.033 a	0.383 ± 0.027 a	0.157 ± 0.027 b	0.166 ± 0.027 b
Hu13130	0.263 ± 0.023 a	0.177 ± 0.018 b	0.146 ± 0.018 b	0.161 ± 0.018 b
HvT02	0.227 ± 0.017 b	0.307 ± 0.014 a	0.299 ± 0.014 a	0.293 ± 0.014 a
Hv1471	0.245 ± 0.016 b	0.317 ± 0.013 a	0.301 ± 0.013 a	0.306 ± 0.013 a
Sb11109	0.311 ± 0.013 a	0.313 ± 0.010 a	0.307 ± 0.010 a	0.268 ± 0.010 b
Sb13129	0.302 ± 0.021 a	0.359 ± 0.017 a	0.337 ± 0.017 a	0.337 ± 0.017 a

^a−c^Values for each strain in the same row followed by different letters are significantly different at *p* ≤ 0.05 according to a Tukey post-hoc test

*Control was synthetic must containing 50 mg/L GABA and 210 mg/L glutamic acid

This effect is likely due to GABA synthesis being an irreversible enzymatic decarboxylation of glutamate (Zhang et al. 2022), catalysed by glutamate decarboxylase (GAD), using pyridoxal phosphate (PLP) as a cofactor (Perpetuini et al. 2020; Yuan et al. 2020) (Fig. 1). Thereafter, GABA aminotransferase (Uga1p) transamination produces succinate semialdehyde (SSA), and SSA dehydrogenase (Uga2p) oxidizes SSA to succinate (Pérez et al. 2022). Therefore, although GABA acts as an intermediary in succinic acid production by glutamate, some yeasts produce less succinic acid in media supplemented with GABA than in media supplemented with glutamic acid. These results suggest that some yeast strains decarboxylate glutamate to produce GABA instead of oxidizing it directly. Nonetheless, it is important to note that in some strains the increase in GABA and glutamic acid encouraged succinic acid production.

## Conclusions

Succinic acid production by non-*Saccharomyces* and *S. cerevisiae* wine yeasts and the influence of GABA and glutamic acid were evaluated. The results show that, considering average values for species, *H. uvarum* and *S. bacillaris* produced significantly more succinic acid than the others, with values higher than 0.3 g/L, while *T. delbrueckii* and *M. pulcherrima* produce amounts of succinic acid like those of *S. cerevisiae*. However, succinic acid production by yeasts is clearly a strain-dependent effect. Nonetheless, a strain-dependent effect was also observed when concentrations of GABA and glutamic acid were increased in the media, where some strains produced higher amounts of succinic acid in the presence of more quantities of GABA or glutamic acid, while others produced less succinic acid, similar to the mixed GABA-glutamic acid treatment.

Consequently, higher succinic acid production by non-*Saccharomyces* yeast in sequential fermentations with *S. cerevisiae* strains —which are also able to produce succinic acid— could represent a risk of stalling MLF because succinic acid can act as an inhibitor of *O. oeni*.

Further research is necessary to understand the mechanisms of promotion and inhibition of succinic acid production by GABA and glutamic acid in yeast. Besides research in synthetic must like this study, further research is needed also in grape must and in real conditions of winemaking, despite its variability. Moreover, it is also necessary to study the best pair of strains in coinoculated fermentations to avoid higher amounts of succinic acid.

## Data Availability

If someone wants to request the data, the first author Rafael Torres-Guardado can be contacted.

## Declarations

This work complies with ethical standards. This article does not contain any studies with human participants or animals performed by any of the authors.
